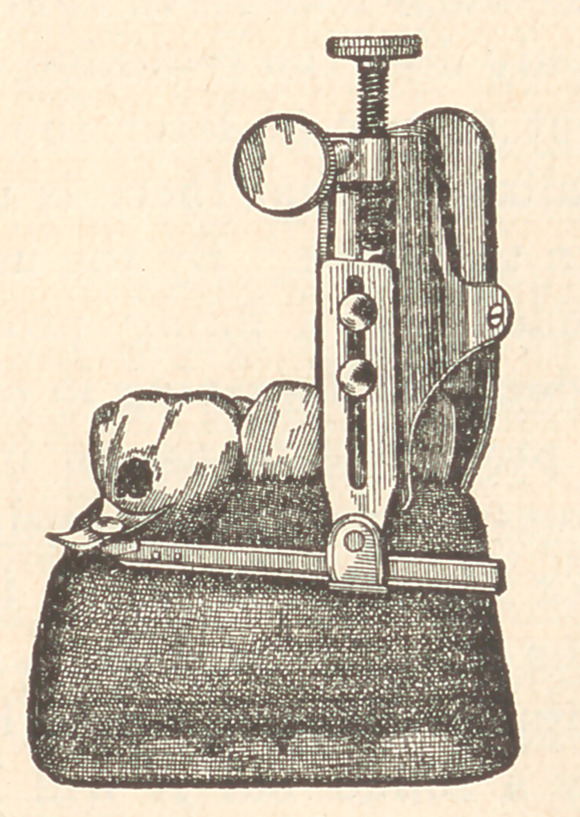# Cervical Cavities and Their Treatment

**Published:** 1894-12

**Authors:** William L. Fish

**Affiliations:** Newark, N. J.


### CERVICAL CAVITIES AND THEIR TREATMENT.[1]

BY WILLIAM L. FISH, D.D.S., NEWARK, N. J.

Mr. President and Gentlemen,—The subject of cervical cavities and their treatment, while not one that we would commonly admit was open to argument, has given rise to so many diverse opinions and methods of operating that further discussion may not seem out of place. In dental operations, as in all other operations, there are certain laws of philosophy, and any deviation from these natural laws will be met by signal failure. That they are violated, in both the manufacture of our instruments and our methods of operating, so far as it relates to the class of cavities with which we have to deal, it is the object of this paper to prove. As practitioners we fully realize that of all cavities with which we have to deal those of a cervical character give us most concern, for in them we may achieve our grandest successes or our most dismal failures. We find that one of the greatest drawbacks to success lies in the difficulty of pushing back the gum, a feature simple of itself, yet conducive to the greatest amount of trouble to patient and operator. The first device used for this purpose was the silk ligature held in the hand of the patient. Then we began to have a class of clamps that have given us fairly good results as far as they have gone. But they had all a vital fault. Being constructed in the form of a spring with unequal arms, they seek the smaller diameter when applied to the tooth, and slip either up or down upon the slightest touch. Also, they are absolutely useless in that form of cavity known as compound cervical approximal, where both cavities run into each other. The use of the paper disk is an impossibility. In the treatment of bicuspids and molars we have been sadly handicapped, as these clamps have been poorly adapted to use upon them. One method that I had brought to my attention this summer deserves a passing mention, as it is the invention of one of our Jersey dentists (a fact that I am not very proud of). This eminent D.D.S. stated that he "never used either silk or clamps of any kind. His office being wired for electricity, he simply attached his cautery. and with the loop burnt the gum away to the desired extent." That, gentlemen, is the nearest approach to the sting of a "hornet" that a Jersey dentist has ever attained. Realizing the great need

---

[1] Read before the Odontological Society of Pennsylvania, October 13, 1894.

for a clamp of this class that would be as nearly as possible universal in its application, I have endeavored to construct one, intended to be used for cavities of both upper and lower jaw, right or left side, taking in molar teeth. There are in its construction none but purely mechanical and positive principles of action, the gum being pushed back by a simple turning of a thumb-screw, the operation being so gradual as to be almost painless to the patient. The method of attaching the clamp to the tooth next the one to be operated upon leaves the latter clear and free for further manipulations. To better enable those present to understand the workings of the clamp, I have made three section models showing its application to the different teeth. You will see at a glance how readily it can be applied. No. 1 shows its application to a lower bicuspid of the right side, the receder arm extending back to the molar tooth so that the gum can be readily forced down upon the neck of the tooth. The clamp may be applied to the other side of the mouth by simply reversing the receder arm in the slide. No. 2 shows the application to the lower centrals and laterals. The narrowness of teeth permits of its being applied to the third tooth, thereby giving ample space for work. No. 3 shows the application to upper centrals and laterals.[1] You will observe the receder is capable of being turned so as to allow its use upon small- or large-necked teeth. In filling teeth in the back of the mouth, where the dam is precluded and where the gum bleeds at the slightest touch, you will find this little device to be invaluable.

A great drawback to the successful filling of these cavities lies

[1] The illustration of the action of this clamp on the molar tooth fully explains the idea, and, therefore, only one is given.—Ed.

in the lack of that power of differentiating between the cavity to be filled with gold and that to be filled with plastics. In the case of a gold filling, I would proceed in the following manner: After the usual preliminaries incident to the use of gold, such as applying the dam, clamp, etc., I would proceed to excavate, taking pains to cut well away all soft or defective enamel, even though it may enlarge the cavity to a considerable extent. In so doing endeavor to so shape the same as to have a symmetrical curvature to the entire line of cavity. Do not have a straight base line to a cavity that may extend entirely across the tooth, for the effect will be to make the work look heavy, inasmuch as the oral cavity is made up entirely of curves. I should avoid in every case the use of the retaining point, which is an abomination, a snare, and a delusion, for by its use more bad results are attained than by any other method now in vogue. Not only does the retaining plug transmit thermal shock to the pulp, but sooner or later the poor deluded patient awakes to the sad realization that there is an ache, notwithstanding a filling remains in the tooth. By the use of a slight undercut these results are avoided and a filling produced that will show the encroachment of decay by loosening in cavity. In the making of an undercut it is preferable to use an inverted cone bur that has not too sharp a point. My reason for using this style of instrument is that the larger diameter of cavity is at the absolute bottom. The result being that the gold as it is forced to the walls in the course of manipulation is bound to hug the floor, while in an undercut made by a round bur it will leave the floor of the cavity, from the fact that it is forced to the side walls and will seek the larger diameter; this larger diameter is at a point one-half the diameter of bur-head from bottom of cavity. It is this fact that causes a filling to "ball up," so to speak. After making my undercut I would level the edge of my cavity from within outward, taking pains to make a clear outline free from any flaws, using to make the same a diamond cone point. A bur will be apt to gouge in and cause trouble. This bevelled edge will tend to lessen the tendency to fracture in the enamel margin, during the condensing and finishing process. I now would proceed to line up my entire cavity with absolutely soft gold, packing it around the entire walls and allowing the ends of the cylinders or ribbons to project a trifle outside of the cavity. After lining throughout in this manner, take semi-cohesive gold and work in upon the layer of soft gold by hand pressure with a rotary motion, which will tend to unite and further condense the first layer. In every case use great care so as not to allow the harder gold to come in contact with the enamel, or allow the instrument to cut through the soft gold. After condensing well I am at liberty to fill in with hard or cohesive gold, and, after a thorough burnishing, finish with any method that may seem most desirable. A filling put in by the foregoing method will be found to stand the test of time. Its very construction making it absolutely tight and the walls and margins free from fracture. In the preparation of a cavity for the reception of any of the soft or plastic fillings, the shape would be the same as that for gold, with the single exception that the bevelled edge should be discarded, and the cavity made as near as possible parallel through the thickness of the enamel. This can best be accomplished by using a right-angled fissure-bur that has the flutes or blades cut on a spiral; the latter form will prevent the bur from chattering, enabling the operator to produce a perfect margin. In the manipulation of alloys I find that the greatest satisfaction is derived from the use of the ball instruments, for the reason that no matter in which direction the pressure is applied it is direct, and the alloy will be found to be more thoroughly condensed than when put in by any other instrument, and the mercury will work to the surface, the ball having the effect of a roller. In finishing be sure and have a surplus of alloy to work down with the burnisher, that little or no mercury may remain in the filling. While the foregoing methods may seem to a casual observer somewhat laborious, a careful study will show that they are based upon certain principles of mechanics which are too often lost sight of in the hurry and worry of a busy professional career.

## Figures and Tables

**Figure f1:**